# Treatment and molecular analysis of bullous pemphigoid with tofacitinib: a case report and review of current literature

**DOI:** 10.3389/fimmu.2024.1464474

**Published:** 2024-10-21

**Authors:** Xiang Li, Lian Zhang, Hongzhi Gu, Wanzhen He, Zhifang Zhai, Mingwang Zhang

**Affiliations:** Department of Dermatology, Southwest Hospital, Army Medical University, Chongqing, China

**Keywords:** bullous pemphigoid, tofacitinib, JAK-STAT pathway, JAK inhibitor, case report

## Abstract

**Background:**

Bullous pemphigoid (BP) is a rare, life-threatening autoimmune blistering disease with pruritus and tension blisters/bullous as the main clinical manifestations. Glucocorticosteroids are the main therapeutic agents for it, but their efficacy is poor in some patients. Tofacitinib, a small molecule agent that inhibits JAK1/3, has shown incredible efficacy in a wide range of autoimmune diseases and maybe a new valuable treatment option for refractory BP.

**Objective:**

To report a case of refractory BP successfully treated with tofacitinib, then explore the underlying mechanism behind the treatment, and finally review similarities to other cases reported in the literature.

**Methods:**

Case report and literature review of published cases of successful BP treatment with JAK inhibitors. The case report describes a 73-year-old male with refractory BP that was successfully managed with the combination therapy of tofacitinib and low-dose glucocorticoids for 28 weeks. Immunohistochemistry and RNA sequencing were performed to analyze the underlying mechanism of tofacitinib therapy. A systematic literature search was conducted to identify other cases of treatment with JAK inhibitors.

**Results:**

Throughout the 28-week treatment period, the patient experienced clinical, autoantibody and histologic resolution. Immunohistochemical analysis showed tofacitinib significantly decreased the pSTAT3 and pSTAT6 levels in the skin lesions of this patient. RNA sequencing and immunohistochemical testing of lesion samples from other BP patients identified activation of the JAK-STAT signaling pathway. Literature review revealed 17 previously reported cases of BP treated with four kinds of JAK inhibitors successfully, including tofacitinib (10), baricitinib (1), upadacitinib (3) and abrocitinib (3).

**Conclusions:**

Our findings support the potential of tofacitinib as a safe and effective treatment option for BP. Larger studies are underway to better understand this efficacy and safety.

## Introduction

Bullous pemphigoid (BP) is a chronic, subepidermal autoimmune disorder that predominantly affects the elderly population. It is characterized by the formation of extensive blisters and severe itching, which can lead to mortality if not managed effectively (1). The primary treatment modalities for BP include glucocorticoids and immunosuppressive agents. However, Long-term administration of these medications is associated with a range of serious adverse effects, and their efficacy varies considerably among patients (2, 3).

In recent years, there has been a discernible shift towards the use of more precise immunotherapies that specifically target extracellular cytokines or pivotal proteins. Notable examples include rituximab, which targets the CD20 protein expressed on B cells; omalizumab, which binds to IgE antibodies; tralokinumab, which targets IL-13; and dupilumab, which inhibits the IL-4Rα receptor. These biological agents have been increasingly utilized in the treatment of BP patients who exhibit resistance to conventional glucocorticoid and immunosuppressive therapies (4–6). However, despite the overall benefits of these therapies, a subset of patients either do not respond or become unresponsive over time, leading to persistently higher mortality rates among BP patients compared to the general population (7, 8).

Therefore, it is imperative to explore novel therapeutic strategies that target intracellular pathways activated by cytokines for future treatment considerations. Tofacitinib, an FDA-approved JAK1/3 inhibitor, is currently used for rheumatoid arthritis and other autoimmune diseases. It can block the intracellular signal transduction of several Th2 and Th17 cytokines, including IL-4, IL-5, IL-13, IL-23, etc., thereby inhibiting the activation of eosinophils, neutrophils, CD4+ T cells, and B cells (9). However, its efficacy in BP has been scarcely documented. This study details the successful use of tofacitinib in treating a refractory BP patient and explores the molecular and transcriptomic mechanisms underlying its therapeutic effects.

## Case report

A 73-year-old male presented to our dermatology clinic with erythema, blisters, erosion, and pruritus on his limbs and trunk. His symptoms began as erythema and papula on the right lower extremity, spreading to involve the trunk and limbs over five years. Previous diagnosis of atopic dermatitis and initial treatment with oral antihistamines and topical glucocorticoids was ineffective at other medical institutions. Recently, the patient developed tense blisters and bullae on erythematous and adjacent normal skin, with erosion and scab formation, leading to our consultation. He had a history of type 2 diabetes, treated with insulin and metformin, and had suboptimal glycemic control.

Skin biopsy showed subepidermal blisters with eosinophilic and neutrophilic infiltration in the dermis (**Supplementary Figure S1A**). Direct immunofluorescence (DIF) revealed linear C3 deposition along the basement membrane zone (BMZ) (**Supplementary Figure S1B**). Serum levels of anti-BP180 and BP230 antibodies were elevated at 143.63 U/ml and 97.15 U/ml, respectively. The patient was diagnosed with BP, with a BPDAI score of 46.5 and a pruritus score of 15. Initial treatment with oral prednisone 40 mg daily and topical halometasone ointment did not control the symptoms, with persistent new blisters and significant itching after one month.

**Figure 1:**
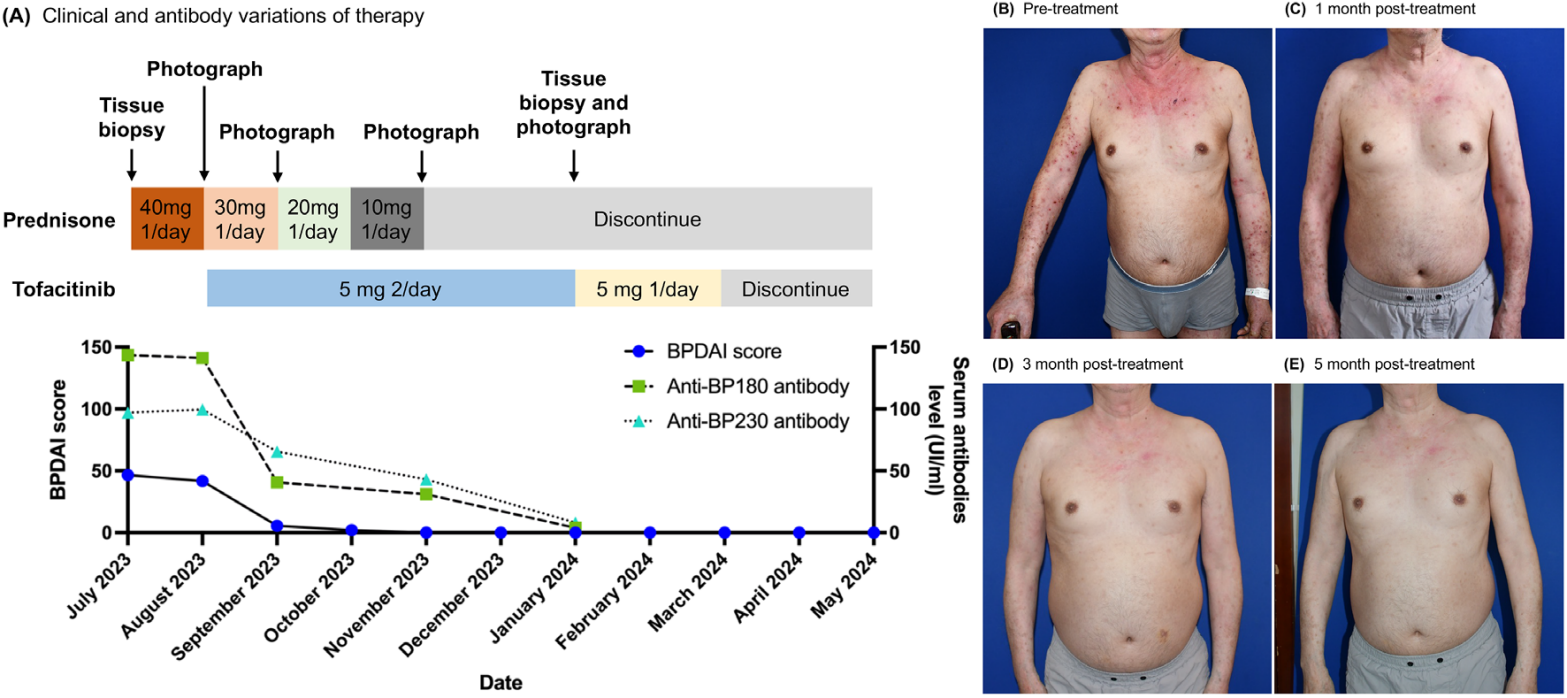
Tofacitinib therapy-induced clinical remission and anti-BP autoantibody reduction in a patient. **(A)** The time course of clinical treatment. The clinical response with therapy is indicated by the change of the BPDAI score and anti-BP180/230 antibody value. **(B–E)** Clinical images of upper extremities and chest & abdomen at baseline and after 1, 3, and 5 months of tofacitinib treatment.

With the patient’s consent and taking into account the patient’s past medical history, we initiated an off-label treatment with tofacitinib 5mg twice daily in combination with prednisone 30mg daily (**Figure 1A**). The treatment outcomes are detailed in the following sections.

## Clinical course

The patient initially presented with a BPDAI score of 41.7 and a PBDAI pruritus score of 13. Following one month of treatment, the BPDAI score markedly improved to 5.6, allowing for a reduction in prednisone to 20mg daily. By the second month, the BPDAI score further reduced to 2, with prednisone dosage lowered to 10mg daily. At the third month, the BPDAI score reached 0, and prednisone was discontinued. Complete remission was achieved at the fifth month accompanied by a reduction of tofacitinib to 5mg daily. Tofacitinib was reduced and eventually stopped by the seventh month. At the report’s compilation, the patient had been free of medication for three months with no signs of relapse (**Figures 1A–E**; **Supplementary Figures S2A–C**). The PBDAI pruritus score dropped to 1 after the initial month and remained at 0 thereafter. Eosinophil counts exhibited a significant decline from 1.55 × 10^9/L (21%) at baseline to 0.12 × 10^9/L (2.5%) one-month post-treatment, stabilizing at 0.13 × 10^9/L (2.6%) during complete remission. Throughout the treatment period, tofacitinib was well-tolerated with no adverse effects noted.

## Anti-BP antibody titer

Anti-BP180 levels decreased from a baseline of 141.29 IU/mL to 40.7 IU/mL at month one and further to 31.02 IU/mL by month three. Anti-BP230 levels also declined, from 99.55 IU/mL at baseline to 65.52 IU/mL at month one and 43.15 IU/mL at month three. By month five, both antibody titers had normalized (**Figure 1A**).

## Immunohistochemistry of phospho-JAK/STAT protein in the patient

**Figure 2:**
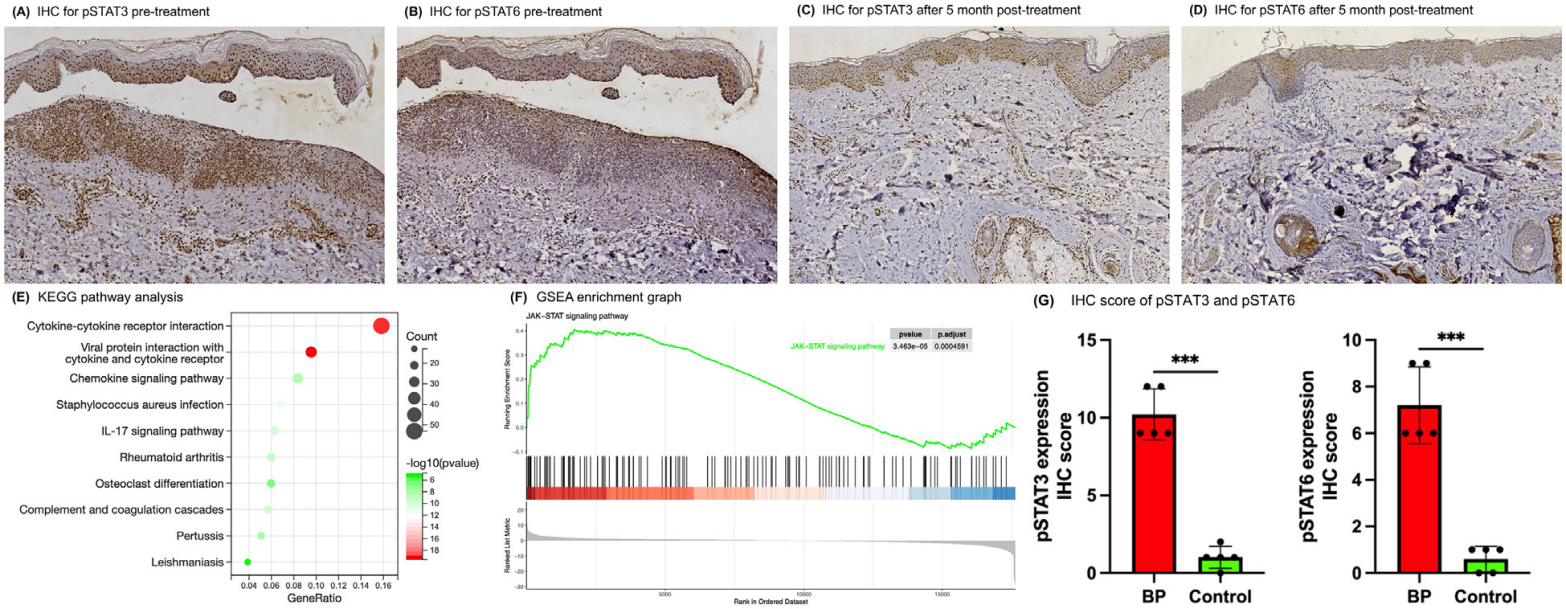
IHC and RNA-seq revealed the activation of the JAK-STAT pathway in skin lesions of BP patients. **(A–D)** IHC analysis of skin lesion biopsies before and post 5-month tofacitinib treatment (original magnification ×100). **(E)** KEGG pathway analysis of up-regulated gene from the RNA-seq data in BP patients compared with health controls. **(F)** GSEA enrichment graphs for the JAK-STAT signaling pathway. **(G)** IHC scores for pSTAT3 and pSTAT6 in skin lesion samples from BP patients versus controls. Error bars indicate mean SD. ***P < 0.001.

After a 5-month treatment period, skin biopsies from previously affected sites demonstrated healing with no subepidermal blisters, fissures, or eosinophilic/neutrophilic infiltration observed on Hematoxylin and eosin (HE) staining (**Supplementary Figure S1C**). Corresponding DIF showed the absence of C3 deposition at the BMZ (**Supplementary Figure S1D**). Immunohistochemical (IHC) analyses further indicated a marked reduction in pSTAT3 and pSTAT6 staining intensities relative to pre-treatment samples (**Figures 2A–D**). Specifically, the IHC scores for pSTAT3 and pSTAT6 decreased from 12 and 9 pre-treatment to 6 and 3 post-treatment, respectively.

## RNA sequencing between BP and controls

RNA sequencing was performed to identify whether the JAK-STAT signaling was activated in BP skin lesions compared with normal controls. Total RNA was extracted from the skin tissue of 3 BP patients and 3 health controls using the TRIzol reagent. Skin lesions of other BP patients were obtained from the department’s archive, and skin lesions of healthy controls were obtained from plastic surgery. The raw sequencing data were visually evaluated by FastQC and filtered by Trim_galore to obtain clean data. HISAT2 was used to map the clean reads to the reference genome, and the quality assessment of mRNA libraries was accomplished by Rseqc. StringTie software was used to analyze transcript abundance. DESeq2 was used for the differentially expressed analysis of mRNAs, and the screening conditions were set with a p-value < 0.05 and | log_2_ Fold Change (FC)| ≥ 1.

A total of 1606 genes were significantly dysregulated between the two groups. Of these, 666 genes were up-regulated, encompassing a mass of cytokines, chemokines, complement-related genes and several matrix metalloproteinases, which have been previously implicated in BP pathogenesis (**Supplementary Figures S3A, B** and **Supplementary Table S1**). KEGG analysis indicated that the up-regulated genes were highly enriched in some pathways, such as the cytokine-cytokine receptor interaction, IL-17 signaling pathway, complement and coagulation cascades pathway and chemokine signaling pathway (**Figure 2E**; **Supplementary Table S2**). Furthermore, to avoid missing some of the genes that were not significantly differentially expressed but biologically important, we performed GSEA analysis on all the genes, and the results showed that activation of the JAK-STAT signaling pathway and several other immune-related pathways in the BP lesion samples (**Figure 2F**; **Supplementary Figure S4**; **Supplementary Table S3**).

## Immunohistochemistry of phospho-JAK/STAT protein in other patients and controls

To further evaluate JAK-STAT activation in BP skin lesions, IHC analysis of pSTAT3 and pSTAT6 was conducted on samples from 5 BP patients and 5 healthy controls. The IHC scores demonstrated markedly elevated for pSTAT3 and pSTAT6 in BP tissues (**Figure 2G**; **Supplementary Figure S5A–D**; **Supplementary Table S4**). Specifically, the mean IHC scores for BP patients were 10.2 ± 1.64 for pSTAT3 and 7.2 ± 1.64 for pSTAT6, contrasting with the control group’s scores of 1 ± 0.71 for pSTAT3 and 0.6 ± 0.55 for pSTAT6.

## Review of literature

A systematic literature search was conducted in order to identify the treatment of BP with JAK inhibitors case reports or series published until the end of April 2024 from the PubMed, Scopus, and Web of Science database, using the following search keyword: (“JAK inhibitors” OR “tofacitinib” OR “Baricitinib” OR “Ruxolitinib” OR “Upadacitinib” OR “Abrocitinib” OR “Peficitinib” OR “Filgotinib” OR “Fedratinib” OR “Bacritinib” OR “Deucravacitinib”) AND (‘‘Bullous Pemphigoid″). The result revealed 17 cases of BP patients treated with JAK inhibitors since 2022 [**Table 1** (10–18)]. Patient ages spanned 10 to 93 years, with the majority exhibiting glucocorticoid resistance, relapse during glucocorticoid reduction, or contraindications to glucocorticoid therapy due to comorbidities, prompting off-label JAK inhibitor use. The treatment demonstrated high efficacy and an acceptable safety profile. Reported JAK inhibitors include tofacitinib (JAK1/3), baricitinib (JAK1/2), upadacitinib (JAK1) and abrocitinib (JAK1).

**Table 1:** Clinical characteristics of previously reported cases of BP treatment with JAK inhibitors.

Cases	Age/gender	Duration	Complication	Previous therapies	Therapeutic regimen	Treatment duration	Outcome	Adverse effects
Xiao et al. (10), 2022	83/Male	2 months	PS, HT	tGC, glycyrrhizin	Baricitinib 4mg qd	6 months	CR	None
Youssef et al. (11), 2022	65/Female	26 months	HPT, HTN, HT, obesity, OA, SpA	Prednisone, doxycycline, niacinamide	Tofacitinib 10mg bid	More than 3 months	CR	Sinus pain
Youssef et al. (11), 2022	76/Male	1 month	DDD, AF	Prednisone, mycophenolate mofetil, dupilumab, rituximab	Tofacitinib 10mg bid, dupilumab	More than 3 weeks	CR	None
Nash et al. (12), 2022	81/Female	4 months	HT, dyslipidemia, OA, endometriosis	Prednisone	Upadacitinib 15mg, prednisone	5 months	CR	None
Fan et al. (13), 2023	67/Male	20 months	None	sGC, minocycline, nicotinamide	Tofacitinib 5mg bid, GC	More than 5 months	CR	None
Fan et al. (13), 2023	10/Male	8 months	DM, osteoporosis	sGC, tGC, mycophenolate mofetil, dupilumab	Tofacitinib 5mg bid, GC	More than 3 months	CR	None
Fan et al. (13), 2023	93/Male	12 months	HT	tGC, minocycline, dupilumab	Tofacitinib 5mg bid, GC	More than 9 months	CR	None
Fan et al. (13), 2023	49/Female	14 months	Cataract	sGC, tGC, minocycline, nicotinamide, dupilumab	Tofacitinib 5mg bid, GC	More than 6 months	CR	None
Fan et al. (13), 2023	75/Male	16 months	None	sGC, minocycline	Tofacitinib 5mg bid, GC	More than 3 months	CR	None
Fan et al. (13), 2023	86/Male	15 months	None	sGC, minocycline, azathioprine	Tofacitinib 5mg bid, GC	More than 7 months	CR	None
Fan et al. (13), 2023	72/Female	22 months	HT	sGC, ciclosporin, azathioprine	Tofacitinib 5mg bid, GC	More than 10 months	CR	None
Gresham et al. (14), 2023	74/Female	more than 10 days	SCCHN	Prednisone, tGC	Upadacitinib 15 mg, prednisone	1 month	Significant improve	None
Li et al. (15), 2023	33/Male	1 week	PS	GC, cyclosporine	Tofacitinib 5mg bid	12 months	CR	None
Lin et al. (16), 2023	61/Female	2 years	HT, osteoporosis,	sGC, methotrexate, tripterygium glycosides, rituximab	Abrocitinib 100mg bid, sGC	6 months	CR	None
Jiang et al. (17), 2024	53/Female	2 years	LSF, PS	Minocycline, GC, cyclophosphamide, cyclosporine, omalizumab	Abrocitinib 100mg qd, GC	5 months	CR	None
Jiang et al. (17), 2024	83/Male	5 months	HT, DU, ST	sGC, tGC, minocycline, nicotinamide, tripterygium glycosides, dupilumab	Abrocitinib 100mg qd, GC	2 months	CR	None
Su et al. (18), 2024	66/Male	2 months	PS	Methylprednisolone, dupilumab	Upadacitinib 15 mg	10 weeks	CR	None
Current case	73/Male	5 years	Type 2 DM	sGC, tGC	Tofacitinib 5mg bid, sGC	7 months	CR	None

HT, hypertension; PS, psoriasis; HPT, Hypothyroidism; HTN, hypercholesterolemia; OA, osteoarthritis; DM, diabetes mellitus; SpA, spondyloarthropathy; DDD, Degenerative disc disease; AF, atrial fibrillation; SCCHN, squamous cell carcinoma of the head and neck; DU, duodenal ulcers; ST, sinus tachycardia; LSF, Lumbar spine fractures; GC, glucocorticoid; tGC, topical glucocorticoid; sGC, systemic glucocorticoid; CR, complete remission.

## Discussion

The JAK/STAT signaling pathway is pivotal in the pathogenesis of immune homeostasis and the development of autoimmune diseases (19). Upon cytokine binding to their receptors, JAKs, including JAK1, JAK2, JAK3, and tyrosine kinase 2 (TYK2), form homodimers intracellularly. These dimers undergo autophosphorylation and subsequently recruit signal transducers and activators of transcription (STAT) proteins. Once phosphorylated, STAT proteins translocate to the nucleus, where they modulate the expression of target genes (20). Juczynska et al. investigated the JAK/STAT pathway in BP and discovered elevated expression levels of all STAT proteins, as well as JAK2 and JAK3, in BP skin lesions (21). They speculated that JAK2 is related to IFN-γ and IL-5 signaling, while JAK3 mainly affects the IL-4 and Th17 axis in BP.

An increasing body of research underscores the significance of Th2 and Th17 cytokines in the etiology of BP. IL-4 and IL-13 are particularly implicated in BP pathogenesis, as they promote Th2 cell differentiation and facilitate B cell immunoglobulin class switching to IgG1 and IgE (22). The involvement of IL-23 and IL-17 in BP progression is supported by their upregulation of proteases that contribute to blister formation, such as matrix metalloproteinase 9 and neutrophil elastase (23). These cytokines primarily exert their biological effects through the downstream transcription factors STAT6 and STAT3. Therefore, the detection of phosphorylation levels of these proteins can reflect the activity of the pathway to a certain extent. This study employed IHC and found that the levels of pSTAT3 and pSTAT6 in BP skin lesions were significantly higher than in healthy individuals. Expression levels were notably reduced following treatment with tofacitinib. Additionally, transcriptome sequencing confirmed the overactivation of the JAK-STAT pathway in BP skin lesions, providing molecular evidence for the first time for the successful treatment of BP with JAK inhibitors.

In this study, tofacitinib was successfully employed to treat a patient with BP who was resistant to glucocorticoids. The patient’s BPDAI score, eosinophil count, and BP180/230 titers rapidly decreased and returned to normal levels, aligning with previous research findings (13). Currently, there have been successful reports of the treatment of BP with four JAK inhibitors: tofacitinib, baricitinib, upadacitinib, and abrocitinib. These agents exhibit different selectivities for JAK kinases, and their inhibitory effects on cytokines differ both *in vitro* and *in vivo* (24, 25). However, only robust clinical trials, including head-to-head studies, will ultimately ascertain whether there are clinically significant differences between these JAK inhibitors in the treatment of BP.

In addition, more research is needed on the safety of JAK inhibitors in patients with BP. Although current case reports on JAK inhibitors in BP show no significant adverse effects, safety in this elderly population must be rigorously assessed, including screening for thromboembolic, malignant, and infectious events before and during treatment (26). JAK inhibitors have garnered attention due to the FDA’s black box warning, the most severe warning for medications. This warning is primarily due to the increased risk of serious cardiovascular events, including myocardial infarction, stroke, and blood clot formation. Additionally, there is a heightened risk of certain cancers and serious infections, particularly in patients with chronic use or pre-existing conditions. Close monitoring and appropriate patient selection are critical to mitigate these adverse outcomes, ensuring that the therapeutic use of JAK inhibitors is both efficacious and safe.

Our research indicates that tofacitinib demonstrates promising therapeutic potential for the management of refractory BP. However, long-term clinical trials are essential to rigorously evaluate the efficacy of tofacitinib in treating BP, as well as to delineate its safety profile over extended periods of use.

## Data Availability

The datasets presented in this study can be found in online repositories. The names of the repository/repositories and accession number(s) can be found below: GSE278926 (GEO).
