# Supplementary material for: Treatment and molecular analysis of bullous pemphigoid with tofacitinib: a case report and review of current literature
Source: Front Immunol. 2024 Oct 21;15:1464474. doi: 10.3389/fimmu.2024.1464474 (PMC11532171; doi:10.3389/fimmu.2024.1464474)
Supplement: Supplementary file 1 [file DataSheet1.docx]

Supplementary Material

## Supplementary Figures


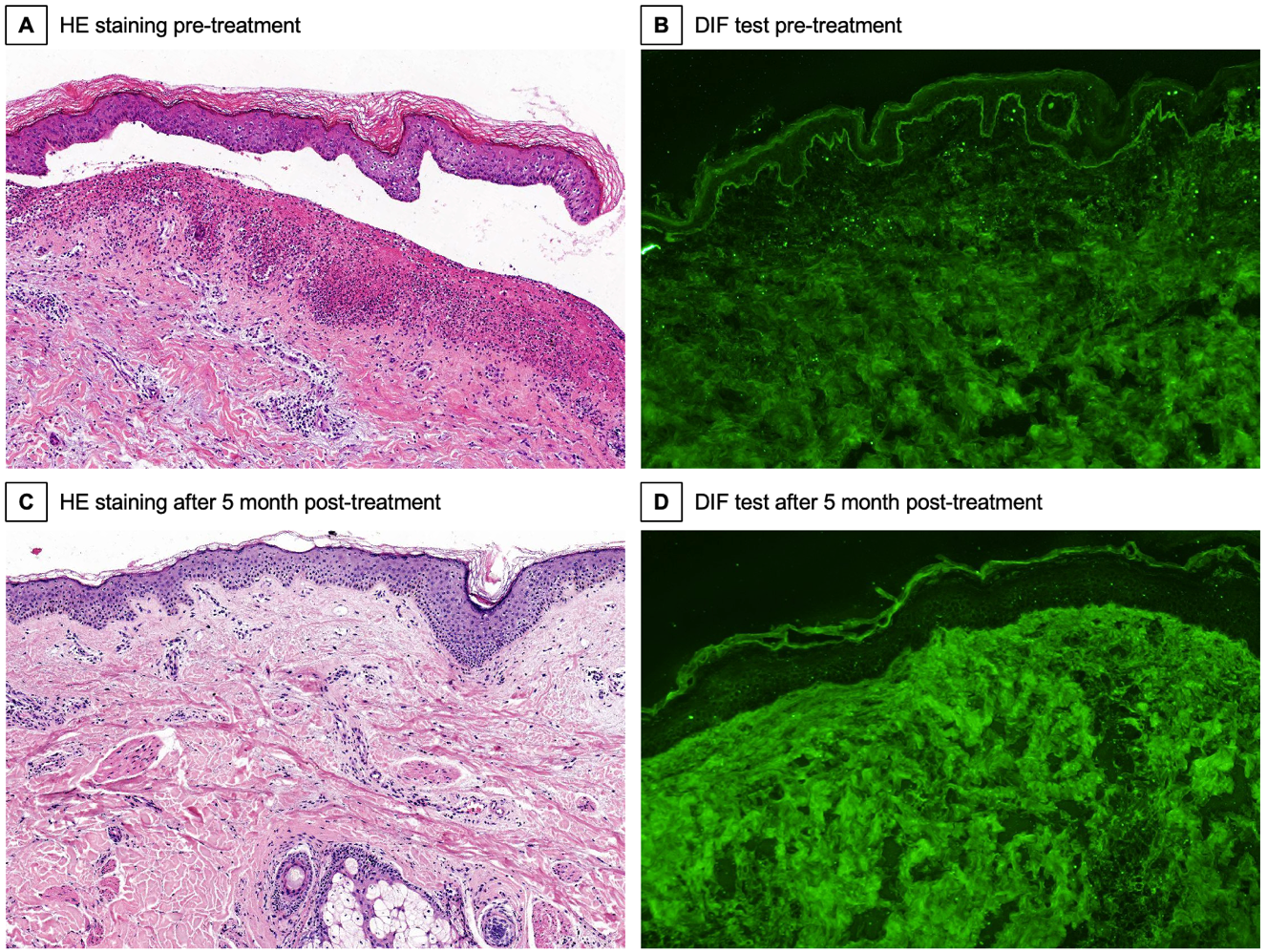


**Supplementary Figure 1.** Histologic and immunologic remission of BP with tofacitinib therapy. (A) and (C) HE staining of the skin lesions obtained at baseline and after 5 months of tofacitinib treatment (original magnification ×100). (B) and (D) DIF test on the sample obtained at baseline and after 5 months of tofacitinib treatment (original magnification ×100).


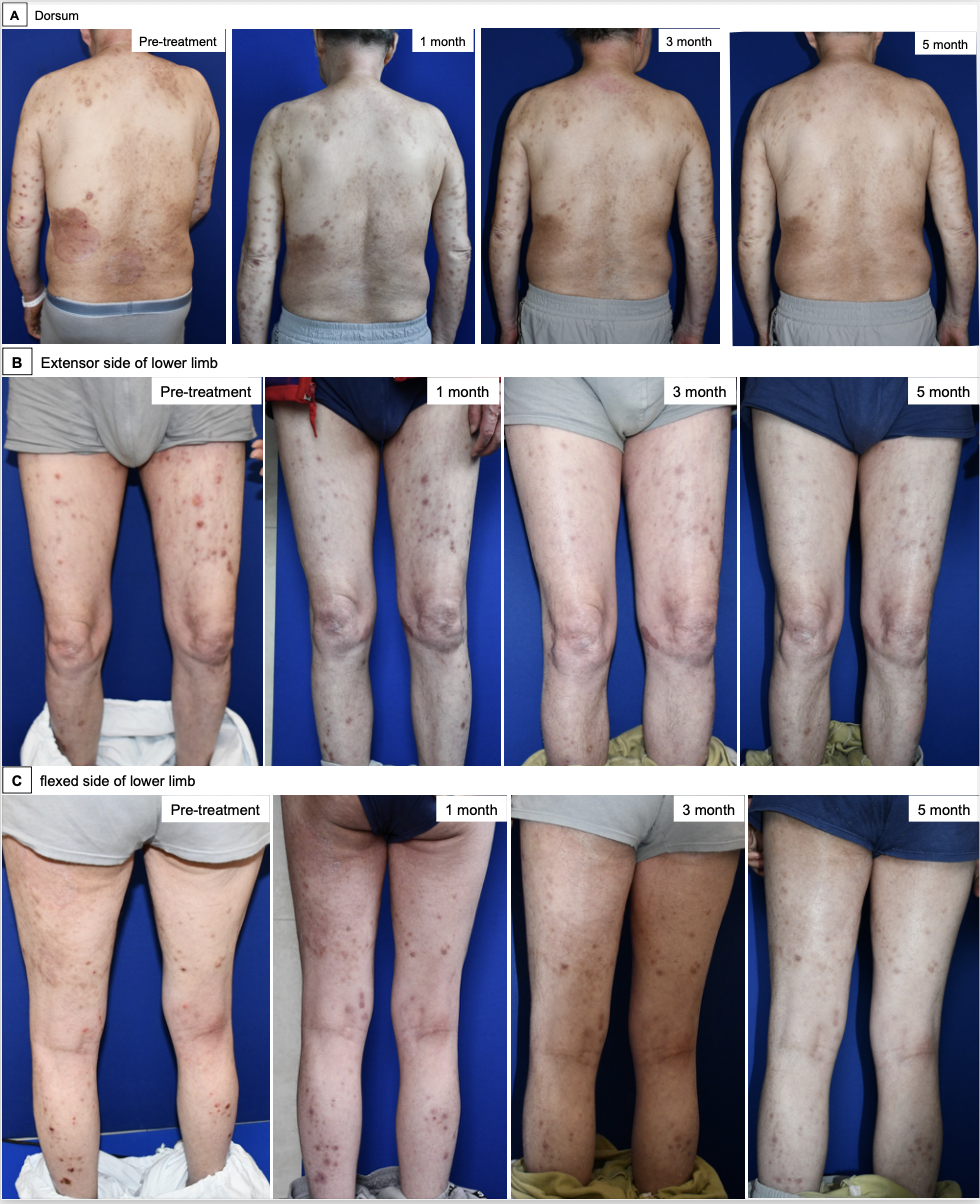


**Supplementary Figure 2.** Additional clinical images before and after 1, 3, and 5 months of treatment with tofacitinib. (A) Photographs of the dorsum at baseline and after 1, 3 and 5 months of treatment with tofacitinib. (B) Photographs of the extensor side of the lower limb at baseline and after 1, 3 and 5 months of treatment with tofacitinib. (C) Photographs of the flexed side of the lower limb at baseline and after 1, 3 and 5 months of treatment with tofacitinib.


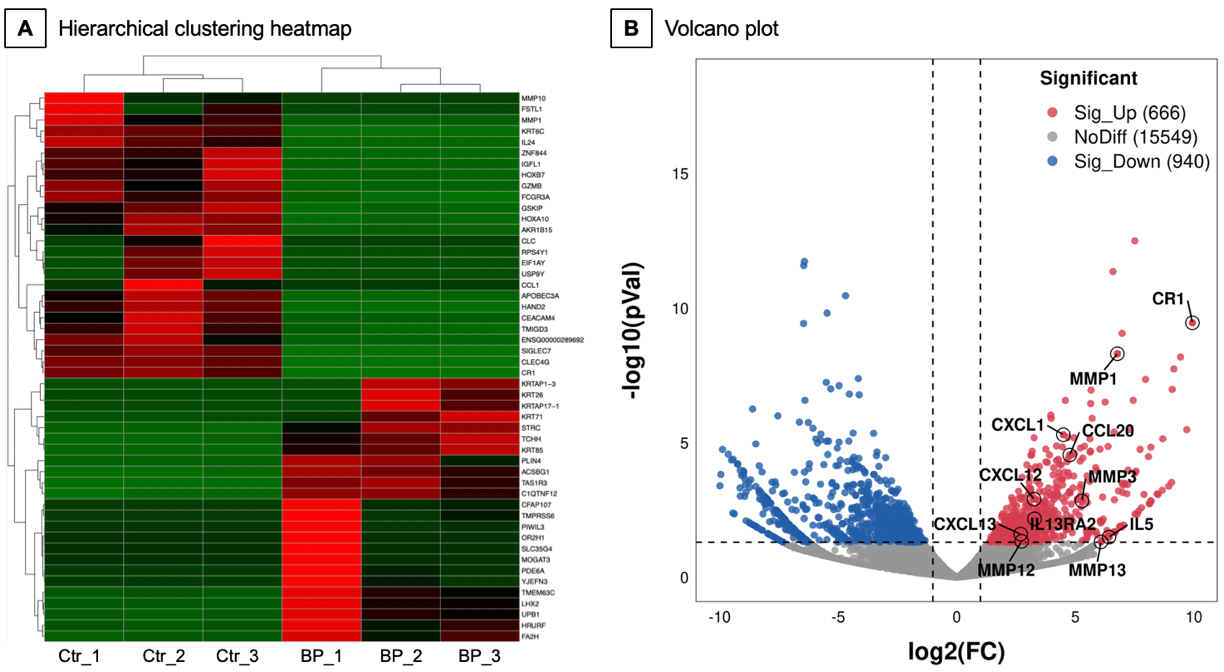


**Supplementary Figure 3.** Gene expression patterns of skin tissue in BP patients and health controls. (A) Hierarchical clustering of top 50 differentially expressed genes. Red indicates up-regulation, and green indicates down-regulation. Columns represent individual samples, and rows represent each gene. (B) Volcano plot of all detected genes. Red dots indicate the up-regulated genes, and blue dots indicate down-regulated genes (|log2 FC| ≥ 1 and p < 0.05).


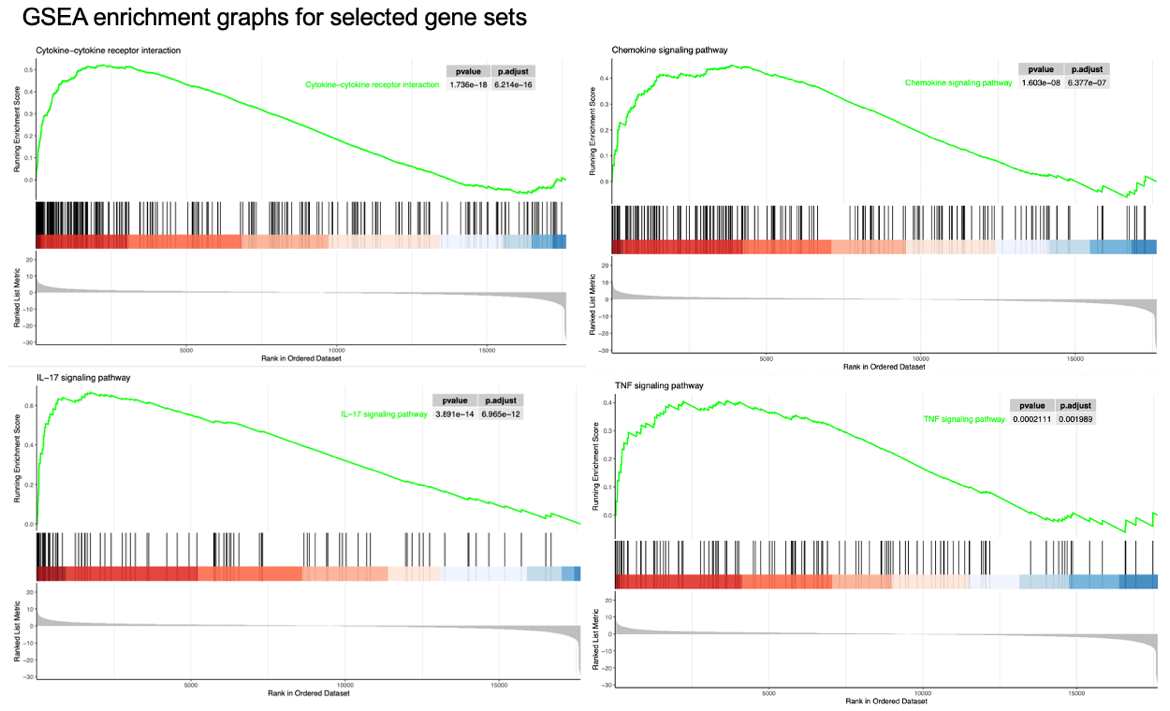


**Supplementary Figure 4.** The GSEA analysis results from RNA-seq data. GSEA enrichment graphs for cytokine-cytokine receptor interaction pathway, chemokine signaling pathway, IL-17 signaling pathway and TNF signaling pathway associated genes in the BP patients compared with health controls.


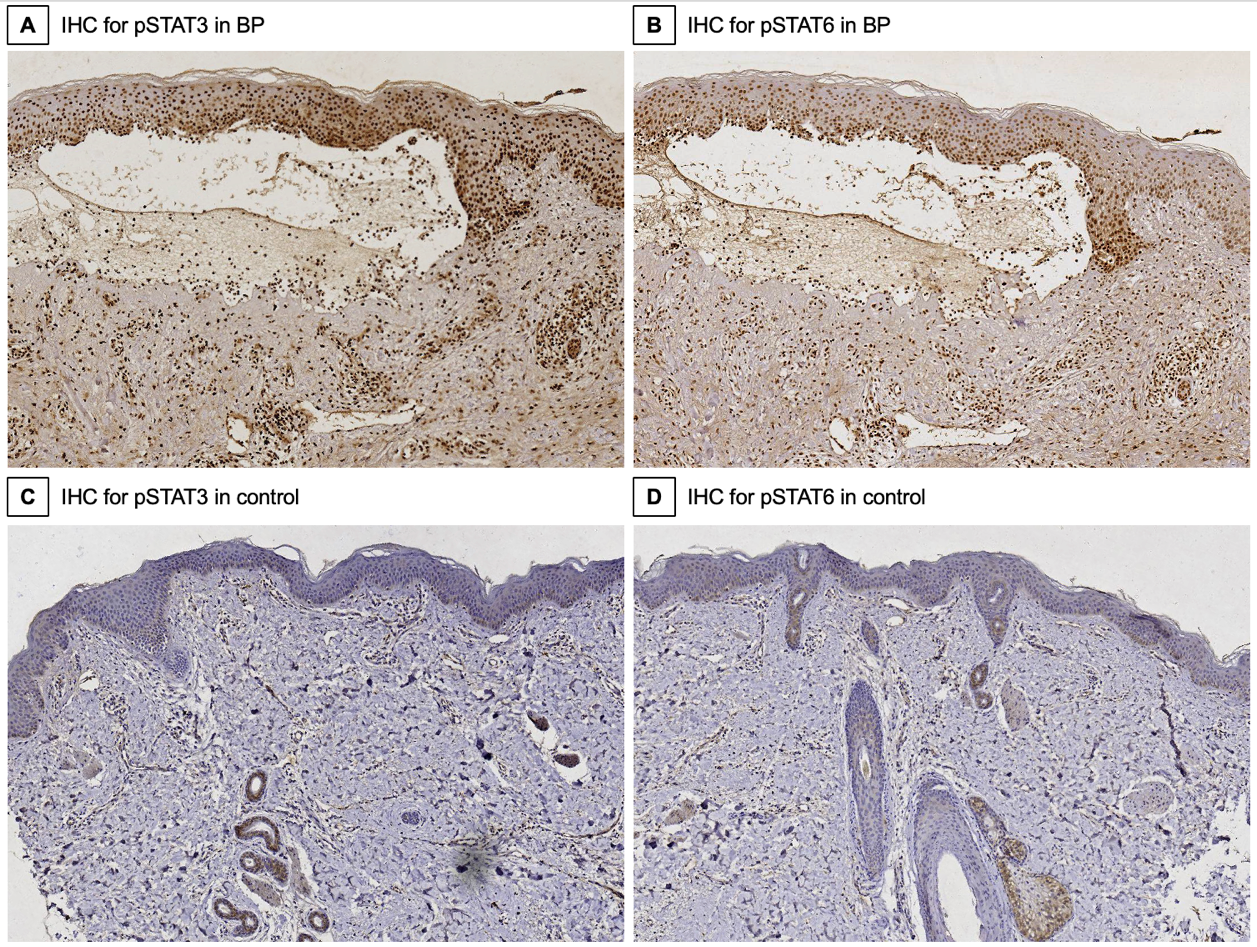


**Supplementary Figure 5.** IHC staining of skin-lesion samples from BP patients and health controls. (A-D) pSTAT3 (left panels) and pSTAT6 (right panels) staining from a biopsy of BP (upper panels) and normal skin (lower panels). These are examples of the histologic images used for quantification (original magnification ×100).

## Supplementary Tables

**Supplementary Table 1.** **Significantly differentially expressed genes in the skin biopsies of BP patients compared with health controls.**

See separate file.

**Supplementary Table 2.** **Complete list of KEGG pathways of up-regulated genes in this study**.

See separate file.

**Supplementary Table 3.** **Full list of GSEA analysis results in this study.**

See separate file.

**Supplementary Table 4. Clinical features of skin biopsies used in RNA-seq and IHC.**

| **Case number** | **Age** | **Sex** | **Location** | **Diagnosis** | **Purpose** |
| --- | --- | --- | --- | --- | --- |
| 1 | 65 | Female | upper arm | Bullous pemphigoid | RNA-seq and IHC |
| 2 | 71 | Male | Forearm | Bullous pemphigoid | RNA-seq and IHC |
| 3 | 53 | Female | Abdomen | Bullous pemphigoid | RNA-seq and IHC |
| 4 | 60 | Female | Forearm | Bullous pemphigoid | IHC |
| 5 | 81 | Male | Chest | Bullous pemphigoid | IHC |
| 6 | 55 | Male | Abdomen | Normal skin | RNA-seq and IHC |
| 7 | 48 | Female | Abdomen | Normal skin | RNA-seq and IHC |
| 8 | 63 | Female | Abdomen | Normal skin | RNA-seq and IHC |
| 9 | 66 | Male | Breast | Normal skin | IHC |
| 10 | 71 | Female | Breast | Normal skin | IHC |
